# In-Hospital Mortality in Patients Receiving Percutaneous Coronary Intervention According to Nurse Staffing Level: An Analysis of National Administrative Health Data

**DOI:** 10.3390/ijerph17113799

**Published:** 2020-05-27

**Authors:** Yunmi Kim, Jiyun Kim

**Affiliations:** 1 School of Nursing, Eulji University, 553 Sanseong-Daero, Sujeong-Gu, Seongnam-Si, Gyeonggi-Do 13135, Korea; kyunm@eulji.ac.kr; 2 School of Nursing, Gachon University, 191 Hambakmoero, Yeonsu-gu, Incheon 21936, Korea

**Keywords:** personnel staffing, nurses, percutaneous coronary intervention, in-hospital mortality

## Abstract

The increasing incidence of ischemic heart disease is concomitantly increasing percutaneous coronary intervention (PCI) treatments. Adequate nurse staffing has enhanced quality of care and this study was conducted to determine the relationship between survival-related PCI treatment and the level of nursing staff who care for patients admitted to receive PCI. National Health Insurance claims data from 2014 to 2015 for 67,927 patients who underwent PCI in 43 tertiary hospitals were analyzed. The relationships of nurse staffing in intensive care units (ICUs) and general wards with survival after PCI were investigated using logistic regression analyses with a generalized estimation model. The in-hospital mortality rate in ICUs was lower in hospitals with first-grade nurse staffing {odds ratio (OR) = 0.33, 95% confidence interval (CI) = 0.23–0.48}, second-grade nurse staffing (OR = 0.55, 95% CI = 0.40–0.77), or third-grade nurse staffing (OR = 0.71, 95% CI = 0.53–0.95) than in hospitals with fifth-grade nurse staffing. Nurse staffing in general wards was not related to in-hospital mortality due to PCI treatment. This study found that nurse staffing in PCI patients requiring short-term intensive care significantly affected patient survival. An understanding of the importance of managing the ICU nursing workforce for PCI treatment is required.

## 1. Introduction

The incidence of ischemic heart disease has been increasing in Korea and medical services were used by 867,540 Korean patients in 2012, which increased to 965,448 in 2016 [1]. The Statistical Yearbook of Health Insurance indicates that the number of patients of acute myocardial infarction (AMI) is also increasing annually; it increased from 69,746 patients in 2011 to 110,328 patients in 2018 [2,3]. Medical expenses have been increasing in Korea in recent years and the medical expense per patient with ischemic heart disease was 6,550,303 KRW (Korean won) in 2012, which increased to 7,291,188 KRW in 2016 [1]. Heart disease as the cause of death for Koreans was third in 2008, and since ranking second in 2014, heart disease has been the second leading cause of death [4]. The in-hospital mortality rate in patients with AMI treated with percutaneous coronary intervention (PCI) in Korea was 3.8% in 2018 [5] and 2.6% between 2011 and 2015 [6].

Increasing the survival probability after ischemic heart disease is very important. Studies in other countries have investigated nurse staffing as an influence on PCI outcomes [7]. Nurses participate in the PCI procedure as a member of the expert group [7] and play an important role in providing patients with education at discharge [8]. Therefore, securing adequate nursing staffing is important for achieving good patient outcomes after the treatment of patients with ischemic heart disease in hospitals.

Economic incentive policies have been developed in Korea to ensure adequate nurse staffing. Founded in 1999, this policy is called the nursing fee differentiation policy (NFDP), and when securing additional nurses per hospital bed, a hospitalization fee was paid to the hospitals as an additional reimbursement from the National Health Insurance Corporation [9,10]. Since the introduction of this policy, the overall nursing staff levels has improved in general wards and intensive care units (ICUs) in Korea [9]. After 2018, the number of beds per nurse, and the standard of paying for additional hospitalization fees, has been gradually changing to patients per nurse [11].

In Korea, nursing staff is known to be significantly related to various patient outcomes such as those for surgery. This study is the first to analyze the relationship between nursing personnel and patient outcomes after PCI, and specifically patient mortality in Korea.

## 2. Methods

### 2.1. Data Source and Sample

**Figure 1:**
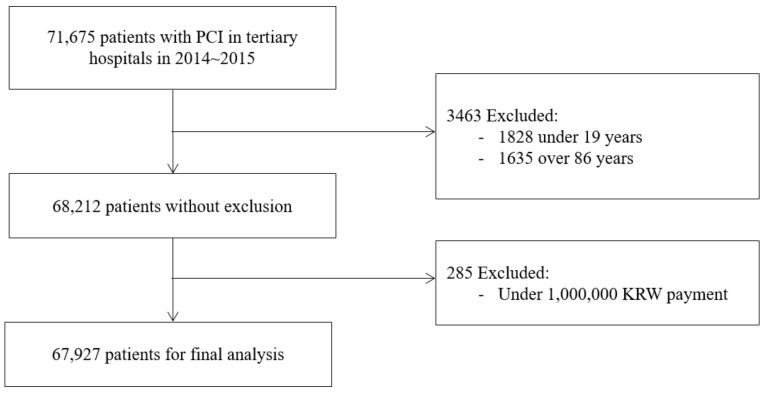
Exclusion and inclusion criteria for the study cohort.

The National Health Insurance (NHI) claims data from 2014 to 2015 from the 43 tertiary hospitals classified by the KDRG (Korean Diagnosis-Related Group) were used to identify PCI. The sampling process is presented in Figure 1.

After a hospital treats a patient, it can charge the reimbursement to the National Health Insurance Corporation (NHIC) within 3 years after the end of treatment [12]. The medical service fee, quality of health care, and adequacy of medical service in hospitals are reviewed by the Health Insurance Review and Assessment Service (HIRA) [13]. HIRA delivers the results to the National Health Insurance Corporation (NHIC), and NHIC reimburses the hospital and builds the dataset [13]. Therefore, it is necessary for the NHIC to have an additional three years to confirm all of the patients treated by the hospital during the given year. In this study, we received a university IRB for data use on March 25, 2019, and we received approval for data use from the NHIC on February 25, 2020.

There were 71,675 patients who received PCI, of which 1828 aged <19 years or ≥85 years were excluded in order to analyze all adult patients other than the very old. We also excluded 285 patients for whom the medical cost was less than 1,000,000 KRW in order to exclude cases of health examinations including biopsy and catheter sonography. The final sample in this study comprised 67,927 patients from 43 tertiary hospitals.

### 2.2. Measurements

#### 2.2.1. Nurse Staffing Level

We investigated the nurse staffing levels and other factors including characteristics of hospitals and patients related to the survival to discharge after PCI.

The nurse staffing level was measured according to the nursing grade, which was categorized based on the bed-to-nurse ratios in general wards and ICUs. According to NFDP, the inpatient reimbursement rates per day were set according to a specified rating based on the number of beds per nurse [14]. Nursing grades were categorized into six levels in general wards and nine levels in ICUs in the tertiary hospitals, with a lower nursing grade indicating a lower bed-to-nurse ratio [14]. The method of measuring nursing staff levels based on the nursing grade in Korea has been explained elsewhere [9,15]. The categories of detailed nursing grade in general wards are as follows: first grade, <2.0 beds per nurse; second grade, ≥2.0~<2.5 beds per nurse; third grade, ≥2.5~<3.0 beds per nurse; fourth grade, ≥3.0~<3.5 beds per nurse; fifth grade, ≥3.5~<4.0 beds per nurse; and sixth grade, ≥4.0 beds per nurse. The classification of nursing grade for ICU is as follows: first grade, <0.5 beds per nurse; second grade, ≥0.5~<0.63 beds per nurse; third grade, ≥0.63~<0.77 beds per nurse; fourth grade, ≥0.77~<0.88 beds per nurse; fifth grade, ≥.0.88~<1.0 beds per nurse; sixth grade, ≥1.0~<1.25 beds per nurse; seventh grade, ≥1.25~<1.5 beds per nurse; eighth grade, ≥1.5~<2.0 beds per nurse; and ninth grade, ≥2.0 beds per nurse.

As only tertiary hospitals that specialize in medical activities with high difficulty for ischemic heart diseases were used in this study, there were only first, second, third, and fifth-grade nursing staff levels in general wards, and only first, second, and third-grade nursing staff levels in ICUs.

#### 2.2.2. Hospital Characteristics

The hospital characteristics consisted of ownership, location, number of PCI procedures, number of beds per physician, and physician in charge of the ICU. Ownership was categorized into public, educational foundation, and medical corporation. A public hospital is funded by the local or central government, an educational-foundation hospital is owned by a university (especially a medical school) and plays an enhanced educational role for medical professionals, while many hospitals in Korea that operate on a not-for-profit basis can be classified as hospitals run by medical corporations.

The location of the tertiary hospitals was categorized based on population size into Seoul, metropolitan (population >1 million), and small city. The number of PCI procedures was also categorized into three groups: ≤999, 1000–1999, and ≥2000. The physician staffing levels were categorized based on the number of beds per physician into three groups: <2.0, <2.3, and ≥2.3. The analytical variables included whether there was a physician in charge of the ICU. 

#### 2.2.3. Patient Characteristics

The patient characteristics consisted of sex, age, type of procedure, disease severity, insurance type, admission path, and admission to the ICU. The sample was divided into six age groups and the following five categories for procedure type: PCI with AMI, PCI without AMI accompanied by stent placement, PCI without AMI not accompanied by stent placement, PCI for arrhythmia therapy, and other PCIs. The disease severity was categorized into three groups according to complications and comorbidities (none, minor, and moderate). Insurance type was categorized into six groups: locally provided policyholder, locally provided dependent, employer-provided policyholder, employer-provided dependent, patient eligible for medical benefit, and dependent eligible for medical benefit.

The medical system in Korea includes Korean health insurance and a medical benefits program. As a compulsory type of social insurance, Korean health insurance covers the entire population living in the country, and for low-income households, the government’s medical benefit program serves as a form of public assistance scheme to assist with self-help by providing medical services [13]. There are two types of health insurance: local health insurance and employer-provided insurance. Each health insurance and medical benefit considers both the policyholder and dependents who are family members of the policyholder.

The admission path was categorized into outpatient clinic and emergency room. Information was also included on whether or not the patient had been admitted to the ICU. The outcome variable of the study was patient death during hospitalization following PCI treatment.

### 2.3. Data Analysis

Descriptive statistics of numbers and percentages were used to quantify hospital and patient characteristics. The chi-square test was used to assess differences in the survival rate after PCI according to the patients and hospital characteristics. Logistic regression applied with a generalized estimation model in order to adjust clustered data was used to analyze the associations between the nursing staff levels and the survival rate after PCI. SAS software (version 9.4, SAS Institute, Cary, NC, USA) was used to analyze the data.

### 2.4. Ethics Statement

Official approval was obtained from the National Health Insurance Corporation to use the data in the present study (official document no. REQ0000034673). This study was exempted from the need to obtain patient consent by the institutional review board (IRB no. EUIRB2019-12) because the National Health Insurance claims data were released data to approved researchers. The data were analyzed at designated big-data laboratories, and it was forbidden to take the data outside the laboratory.

## 3. Results

The hospital characteristics are presented in Table 1. There were eight (18.6%), 30 (69.8%), and five (11.6%) hospitals owned by the public, educational foundations, and medical corporations, respectively. The survival rate was highest in the hospitals owned by medical corporations (99.4%; χ^2^ = 48.72, *p* < 0.001). There were 17 (39.6%), 13 (30.2%), and 13 (30.2%) hospitals located in Seoul, metropolitan areas, and small cities, respectively, and the survival rate was highest in the hospitals in Seoul (99.2%; χ^2^ = 69.84, *p* < 0.001). Twelve hospitals with more than 2000 procedures per year constituted 27.9%, while the survival rate was highest in the hospitals with the largest number of PCI procedures (χ^2^ = 102.44, *p* < 0.001). There were 13 (30.2%), nine (20.9%), and 21 (48.8%) hospitals that had <2.0, <2.3, and >2.3 number of beds per physicians, respectively, and the survival rate increased with the number of beds per physicians (χ^2^ = 75.21, *p* < 0.001). There were 40 (93.0%) hospitals that had a physician in charge of the ICU, and the survival rate after the PCI procedures was not significantly affected by whether or not the hospital had a physician in charge of the ICU. The nursing staff levels were categorized as first grade in seven (16.3%) of the ICUs, second grade in 23 (53.5%), third grade in 11 (25.6%), and fifth grade in two (4.7%). The survival rate after PCI was significantly higher in ICUs with more nurses per bed (χ^2^ = 123.21, *p* < 0.001). There were six (14.0%) hospitals with first-grade nursing staff in general wards, 19 (44.2%) with second-grade nursing staff, and 18 (41.9%) with third-grade nursing staff. The survival rate after PCI was significantly higher in the general wards with more nurses per bed (χ^2^ = 88.89, *p* < 0.001).

The overall patient characteristics and the survival rate after PCI according to patient characteristics are presented in Table 2. There were 69.9% male patients and 30.1% female patients. Those aged 70–84 years constituted the majority of the sample (32.8%), followed by those aged 60–69 years (27.7%) and 50–59 years (24.0%). Most of the patients underwent a PCI without AMI with stent placement (41.6%), followed by PCI with AMI (25.8%), other PCI (15.5%), PCI for arrhythmia therapy (12.8%), and PCI without AMI without stent placement (4.4%). Most of the patients (73.6%) had no complication or comorbidity, 63.5% of patients were admitted via outpatient clinics and 55.6% were admitted in ICUs.

Most of the patients had insurance for employer-provided dependents (40.1%), followed by that for employer-provided policyholders (25.0%), locally provided policyholders (22.2%), locally provided dependents (7.5%), patients eligible for medical care (4.5%), and dependents eligible for medical care (0.6%).

The survival rate after PCI was higher in males (χ^2^ = 13.86, *p* < 0.001), younger patients (χ^2^ = 464.62, *p* < 0.001), those undergoing PCI for arrhythmia therapy (χ^2^ = 576.12, *p* < 0.001), those with lower disease severity (χ^2^ = 479.45, *p* < 0.001), health insurance policyholders (χ^2^ = 188.20, *p* < 0.001), those admitted via outpatient clinics (χ^2^ = 836.29, *p* < 0.001), and those not admitted to ICUs (χ^2^ = 562.84, *p* < 0.001).

The in-hospital mortality rates according to the nurse staffing status are presented in Table 3. In the multivariate model, the probability of in-hospital mortality in ICUs was lower in hospitals with first-grade nursing staff {odds ratio (OR) = 0.33, 95% confidence interval (CI) = 0.23–0.48}, second-grade nursing staff (OR = 0.55, 95% CI = 0.40–0.77), or third-grade nursing staff (OR = 0.71, 95% CI = 0.53–0.95) than in hospitals with fifth-grade nursing staff.

## 4. Discussion

The present study found that in-hospital mortality was related to the nurse staffing in ICUs. The probability of survival was higher in patients receiving PCI when an adequate nursing staff level was provided in ICUs. There were first, second, third, and fifth grade nurses in the ICUs in the data analyzed in this study, and it was confirmed that in-hospital mortality was significantly low when nursing staff were better than fifth grade. However, the nursing staff in the general wards were not related to the in-hospital mortality of patients receiving PCI. According to NFDP, nursing grades in tertiary hospitals included first grade to sixth grade. Since the hospitals used in the results of the study only had first to third-grade nursing staff, the overall nursing grade was excellent, so the relationship between nursing grade and in-hospital mortality was not confirmed. 

Nursing personnel is an important factor related to in-hospital mortality for patients receiving PCI. Nurses provide care based on the evidence before and after a PCI procedure [16] as well as on the recognition of the patient’s symptoms and preparing quickly for treatment decisions [16,17]. Previous studies have provided descriptions of the nursing activities related to PCI procedures [18] and have summarized nursing activities as symptom recognition, treatment decisions, peri-PCI care, discharge planning, and secondary prevention [18]. As a secondary prevention activity to maintain good health after PCI, it is important for nurses to plan and monitor exercise levels [19]. Nurses reduce the uncertainty and fears that patients experience during physical activity in the early post-PCI phase [20]. Adequate nursing staff will prevent missing essential care [21] and will affect the delivery of nursing care for recovery and reduction of mortality in patients with PCI.

This study has revealed from an analysis of administrative data that nursing staff levels are related to the in-hospital mortality rate in patients receiving PCI procedures. A few previous studies performed in other countries have provided data on 30-day survival after PCI [22]. One study analyzed 30-day mortality in PCI-treated patients in a tertiary care center from 2009 to 2011, with 42% of deaths associated with PCI procedures [22]. The cases of 30-day mortality were due to the PCI-related complications of stent thrombosis, bleeding, coronary dissection, and renal failure [22]. The cases of 30-day mortality that were not directly related to PCI treatment were due to lifestyle management and medication management for post-treatment medical care [23]. Age and clinical characteristics such as cardiogenic shock and dyslipidemia also affect 30-day mortality in patients receiving PCI [24]. The present study selected in-hospital mortality rather than 30-day mortality as the outcome variable with the aim of avoiding the inclusion of deaths for reasons other than the PCI procedure and since patients undergoing the PCI procedure experience short hospitalizations.

There were some limitations to this study. First, the study had a cross-sectional design and so it was not possible to confirm the causality of the relationship between nurse staffing and in-hospital mortality in patients receiving PCI. Second, when measuring nursing staff levels, the number of beds per nurse was measured, and not the patients per nurse. This is because nursing grades were measured on the basis of hospital beds at the time of the construction of claims data in 2014 and 2015. In 2019, nursing grades in general hospitals, hospitals, dental hospitals, and oriental hospitals have been changed to be categorized based on the number of patients per nurse [11]. In future, it would be possible to investigate the nursing staff levels with the patient to nurse ratio. Notwithstanding these limitations, this study has provided evidence on the important role that nursing staff can play in reducing the in-hospital mortality rate in patients receiving PCI.

## 5. Conclusions

The probability of patient death after PCI reduced when the nursing grade improved to third, second, and first grades when compared to fifth grade in ICUs after controlling for hospital characteristics and patient characteristics. The nursing staff level is important for patient survival after PCI treatment and it should be improved in ICUs in order to ensure patient safety.

## Figures and Tables

**Table 1:** Hospital characteristics including nursing staff levels.

Variable	Category	Hospitals *N* (%)	Patients *N* (%)	Survival (%)	χ^2^	*p*
Ownership	Public	8 (18.6)	13,927 (20.5)	98.6	48.72	<0.001
	Educational foundation	30 (69.8)	41,390 (60.9)	98.7		
	Medical corporation	5 (11.6)	12,610 (18.6)	99.4		
Location	Seoul	17 (39.6)	29,845 (43.9)	99.2	69.84	<0.001
	Metropolitan	13 (30.2)	21,170 (31.2)	98.5		
	Small city	13 (30.2)	16,912 (24.9)	98.5		
Number of PCI procedures	62–999	13 (30.2)	9470 (13.9)	98.1	102.44	<0.001
	1000–1999	18 (41.9)	24,337 (35.8)	98.5		
	≥2000	12 (27.9)	34,120 (50.2)	99.2		
Number of beds per physician	<2.0	13 (30.2)	27,228 (40.1)	99.2	75.21	<0.001
	<2.3	9 (20.9)	10,901 (16.1)	98.8		
	≥2.3	21 (48.8)	29,798 (43.9)	98.4		
Physician in charge of the ICU	No	3 (7.0)	1702 (2.5)	98.5	1.05	0.305
	Yes	40 (93.0)	66,225 (97.5)	98.8		
ICU nurse staffing	First grade	7 (16.3)	20,696 (30.5)	99.5	123.21	<0.001
	Second grade	23 (53.5)	32,036 (47.2)	98.6		
	Third grade	11 (25.6)	13,744 (20.2)	98.3		
	Fifth grade	2 (4.7)	1451 (2.1)	97.7		
General-ward nurse staffing	First grade	6 (14.0)	15,440 (22.7)	99.5	88.89	<0.001
	Second grade	19 (44.2)	27,781 (40.9)	98.7		
	Third grade	18 (41.9)	24,706 (36.4)	98.5		

**Table 2:** Patient characteristics.

Variable	Category	Patients *N* (%)	Survivors *N* (%)	χ^2^	*p*
Sex	Male	47,489 (69.9)	46,967 (98.9)	13.86	<0.001
	Female	20,438 (30.1)	20,144 (98.6)		
Age, years	20–29	1038 (1.5)	1035 (99.7)	464.62	<0.001
	30–39	2281 (3.4)	2277 (99.8)		
	40–49	7205 (10.6)	7172 (99.5)		
	50–59	16,278 (24.0)	16,188 (99.5)		
	60–69	18,824 (27.7)	18,691 (99.3)		
	70–84	22,301 (32.8)	21,748 (97.5)		
Type of procedure	PCI with AMI	17,534 (25.8)	17,060 (97.3)	576.12	<0.001
	PCI without AMI accompanied by stent placement	28,236 (41.6)	28,101 (99.5)		
	PCI without AMI not accompanied by stent placement	2982 (4.4)	2949 (98.9)		
	PCI for arrhythmia therapy	8667 (12.8)	8665 (100.0)		
	Other PCIs	10,508 (15.5)	10,336 (98.4)		
Disease severity	No complication or comorbidity	50,018 (73.6)	49,691 (99.4)	479.45	<0.001
	Minor complication or comorbidity	12,990 (19.1)	12,632 (97.2)		
	Moderate complication or comorbidity	4919 (7.2)	4788 (97.3)		
Insurance type	Locally provided policyholder	15,101 (22.2)	14,951 (99.0)	188.20	<0.001
	Locally provided dependent	5073 (7.5)	4987 (98.3)		
	Employer-provided policyholder	16,988 (25.0)	16,930 (99.7)		
	Employer-provided dependent	27,255 (40.1)	26,798 (98.3)		
	Patient eligible for medical benefit	3078 (4.5)	3024 (98.3)		
	Dependent eligible for medical benefit	432 (0.6)	421 (97.5)		
Admission path	Outpatient clinic	43,143 (63.5)	43,020 (99.7)	836.29	<0.001
	Emergency room	24,784 (36.5)	24,091 (97.2)		
Admission to the ICU	No	37,767 (55.6)	37,648 (99.7)	562.84	<0.001
	Yes	30,160 (44.4)	29,463 (97.7)		

**Table 3:** Association of nurse staffing with in-hospital mortality due to PCI.

Variable	Category	OR	(95% CI)	*p*
ICU nursing staff (ref = fifth grade)	First grade	0.33	(0.23–0.48)	<0.001
	Second grade	0.55	(0.40–0.77)	<0.001
	Third grade	0.71	(0.53–0.95)	0.022
General-ward nursing staff (ref = third grade)	First grade	1.13	(0.63–2.03)	0.682
	Second grade	1.00	(0.73–1.35)	0.985

The model was adjusted while controlling for hospital characteristics (ownership, location, number of PCI procedures, number of beds per physician, and physician in charge of the ICU) and patient characteristics (sex, age, type of procedure, disease severity, insurance type, admission path, and admission to the ICU).
